# Generation of a Hetero Spin Complex from Iron(II) Iodide with Redox Active Acenaphthene-1,2-Diimine

**DOI:** 10.3390/molecules26102998

**Published:** 2021-05-18

**Authors:** Dmitriy S. Yambulatov, Stanislav A. Nikolaevskii, Mikhail A. Kiskin, Kirill V. Kholin, Mikhail N. Khrizanforov, Yulia G. Budnikova, Konstantin A. Babeshkin, Nikolay N. Efimov, Alexander S. Goloveshkin, Vladimir K. Imshennik, Yurii V. Maksimov, Evgeny M. Kadilenko, Nina P. Gritsan, Igor L. Eremenko

**Affiliations:** 1 N. S. Kurnakov Institute of General and Inorganic Chemistry, Russian Academy of Sciences, 31 Leninsky Prosp, 119991 Moscow, Russia; mkiskin@igic.ras.ru (M.A.K.); bkonstantan@yandex.ru (K.A.B.); nnefimov@yandex.ru (N.N.E.); ilerem@igic.ras.ru (I.L.E.); 2 Arbuzov Institute of Organic and Physical Chemistry, FRC Kazan Scientific Center of RAS, Arbuzov Str. 8, 420088 Kazan, Russia; kholin@iopc.ru (K.V.K.); khrizanforov@iopc.ru (M.N.K.); yulia@iopc.ru (Y.G.B.); 3 Nesmeyanov Institute of Organoelement Compounds, 119991 Moscow, Russia; golov-1@mail.ru; 4 N. N. Semenov Institute of Chemical Physics, Russian Academy of Sciences, Kosygina Str. 4, 119991 Moscow, Russia; vladim_imshennik@mail.ru (V.K.I.); maksimov@chph.ras.ru (Y.V.M.); 5 V.V. Voevodsky Institut of Chemical Kinetics and Combustion, SB RAS, 3 Institutskaya str., 630090 Novosibirsk, Russia; e.kadilenko@g.nsu.ru (E.M.K.); gritsan@kinetics.nsc.ru (N.P.G.); 6 Department of Natural Sciences, Novosibirsk State University, 2 Pirogova Str., 630090 Novosibirsk, Russia

**Keywords:** iron(II) complex, α-diimine, BIAN, cyclic voltammetry, molecular structure, magnetic measurements

## Abstract

The reaction of the redox active 1,2-bis[(2,6-diisopropylphenyl)imino]acenaphthene (dpp-BIAN) and iron(II) iodide in acetonitrile led to a new complex [(dpp-BIAN)Fe^II^I_2_] (**1**). Molecular structure of **1** was determined by the single crystal X-ray diffraction analysis. The spin state of the iron cation in complex **1** at room temperature and the magnetic behavior of **1** in the temperature range of 2–300 K were studied using Mossbauer spectroscopy and magnetic susceptibility measurements, respectively. The neutral character of dpp-BIAN in **1** was confirmed by IR and UV spectroscopy. The electrochemistry of **1** was studied in solution and solid state using cyclic voltammetry. The generation of the radical anion form of the dpp-BIAN ligand upon reduction of **1** in a CH_2_Cl_2_ solution was monitored by EPR spectroscopy.

## 1. Introduction

The synthesis of molecular compounds with controlled physicochemical properties [1] is a great task in modern chemistry. For more than 20 years after the discovery of magnetic bistability in the Mn_12_OAc cluster [2], a large number of new complexes with magnetic behavior depending on temperature and applied magnetic field have been synthesized [3,4,5,6]. These compounds, which exhibit a slow relaxation of magnetization below a certain temperature, are called single molecule magnets (SMMs). Molecular materials based on SMMs can be considered as a part of future and even modern electronic devices (e.g., high-density information storage) [7,8,9,10,11].

Initially, the search for SMMs was focused on the synthesis of exchange-coupled clusters based on high-spin 3d metal ions or containing ions of 3d and 4f elements [12,13,14,15]. In 2010, Long and co-workers synthesized a series of mononuclear Fe(II) complexes exhibiting slow magnetic relaxation and named such compounds single-ion magnets (SIMs) [16]. Since this achievement, a large series of SIMs based on mononuclear complexes of 3d metal ions (Fe(I)/(II), Mn(II)/(III), Co(I)/(II), and Ni(I)/(II)) have been reported, and their magnetic properties have been thoroughly investigated [17,18,19,20,21]. Coordination environment, as well as the valence state of the ion, were found to be the determining factors for the realization of SIM properties [6].

There are many examples of iron(II) complexes possessing SMM or SIM behavior [4,6,16,22,23,24,25,26], but very rare examples of metal complexes are known that demonstrate both pronounced redox activity and SIM behavior [5]. The combination of non-innocent ligands based on α-diimines [27] and a redox-active iron ion has led to complexes exhibiting spin-crossover phenomenon [28,29] and promising catalytic activity in hydrogenations of olefins [30], hydrosilylation of aldehydes or carbonyl compounds [31,32,33], polymerization of ethylene [34] and styrene [35], and water reduction [36]. Among the existing α-diimine iron dihalides, the most studied complexes are chlorides (over a hundred deposited structures in CCDC) and bromides (about two dozen structures), and much less is known about iodides [37,38,39]. To our best knowledge, there are no data on α-diimine complexes of iron dihalides demonstrating the SMM/SIM behavior, although, according to theoretical predictions [17] and a series of experimental observations [6,40], heavier main group donor atoms in the first coordination sphere are able to minimize vibronic coupling and increase the spin-orbit coupling. Both of these factors have a positive impact on SIM behavior activation. We have recently shown that replacement of two pyridine ligands with dpp-BIAN in cobalt(II) iodide resulted in an increase in the zero-field splitting parameter (*D*), which is valuable for SIM [41].

Among α-diimines, the dpp-BIAN is of particular interest owing to its special features: (1) lone electron pairs of nitrogen atoms are shielded by bulky ^i^Pr substituents, which prevents the formation of associates; (2) the conformationally rigid diimine system prevents reach conformational dynamic, which facilitates the isolation of molecular systems in the form of single crystals; (3) the well-known exceptional feature of the dpp-BIAN is its ability to accept and delocalize up to four extra electrons into the π system (diimine plus naphthalene) [42]. The synthesis of coordination compounds based on the non-innocent dpp-BIAN ligand and a salt consisting of a redox-active cation (Fe^2+^) and anion (I^−^) is a challenging task. Such a combination of redox-active species in one molecule can hypothetically leads to multistep redox-activity or even to redox-isomerism: [(dpp-BIAN)Fe^II^I_2_] ↔ [(dpp-BIAN)^•−^ Fe^III^I_2_].

Herein, we present the synthesis of the iron diiodide complex [(dpp-BIAN)Fe^II^I_2_] (**1**), multilateral studies of its composition, structure, physicochemical properties, as well as the study of the possibility of obtaining a hetero-spin complex on its basis.

## 2. Results and Discussion

### 2.1. Synthesis and Characterization

**Scheme 1:**
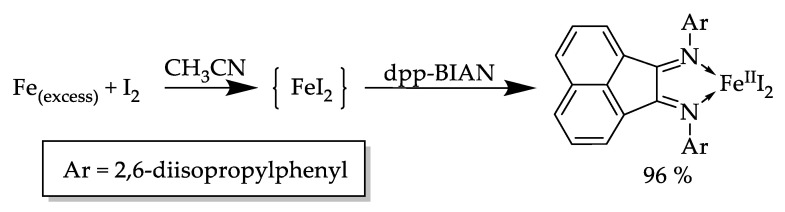
Synthesis of complex **1**.

The dpp-BIAN was synthesized according to a known procedure [43]. Complex [(dpp-BIAN)Fe^II^I_2_] (**1**) was synthesized from iron(II) iodide and dpp-BIAN in acetonitrile solution in a sealed evacuated ampoule (Scheme 1). Anhydrous FeI_2_ is extremely air-sensitive compound, thus, it was synthesized and used in situ to prevent oxidation of Fe^2+^ (commercial anhydrous FeI_2_ undergoes oxidation even when stored in a glove box). The synthesis of iron diiodide in a Schlenk tube with a Teflon stopcock in CH_3_CN is a very simple procedure and takes about 30 min. Complex **1** was isolated from the mother liquor as brown crystals in an excellent yield of 96%. Although FeI_2_ is air-sensitive, compound **1** is quite stable in the presence of oxygen and air moisture in solid.

In the IR spectrum of compound **1** (see Supplementary Figure S1), there is no absorption band associated with the dpp-BIAN radical anion (1510–1515 cm^−1^ [39,44]). The presence of the dpp-BIAN neutral form in complex **1** is indicated by the absorptions with maxima at 1627 and 1645 cm^−1^, which are lower than for the C=N stretching vibrations in free dpp-BIAN (1671, 1652 cm^−1^) [43] and are similar to the C=N stretching frequencies in [(dpp-BIAN)Fe^II^Br_2_] (1643, 1614 cm^−1^) [32]. The band-shift to the lower wavenumbers indicates the coordination of two dpp-BIAN α-diimine nitrogen atoms.

### 2.2. Molecular Structure

**Figure 1:**
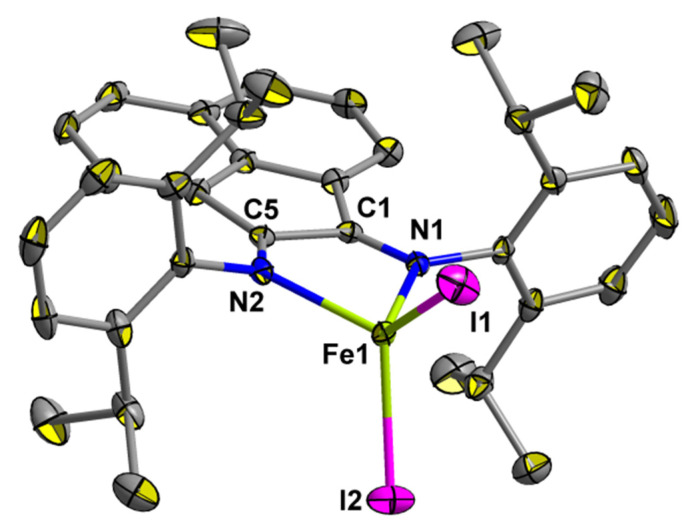
Molecular structure of **1**. Thermal ellipsoids are drawn at 50% probability level. Hydrogen atoms and solvent molecule are omitted for clarity.

Molecular structure for **1** was determined by single-crystal X-ray diffraction at 100 and 280 K (Figure 1). Crystal data and refinement statistics for **1** are summarized in Table 1, while selected bond lengths and angles are presented in Table 2.

Complex **1** crystallizes as solvate with one molecule of CH_3_CN (**1**·CH_3_CN). The coordination environment (experiment at 100 K) of Fe atom (FeN_2_I_2_ chromophore) corresponds to a distorted tetrahedron. In comparison with [(dpp-BIAN)Fe^II^Cl_2_] [31] and [(dpp-BIAN)Fe^II^Br_2_] [32], the angle Hal−Fe−Hal′ for **1** (113.44(2)°) is slightly lower than for the values found in the independent complexes of chloride (122.40(5)° and 118.78(5)°) and bromide analogues (118.92(6)° and 117.09(6)°) for chloride (122.40°), thus, the larger ionic radius of iodine, bulky isopropyl substituents make a greater contribution to the formation of that angle. The value of the Hal−Fe−Hal′ angle does not affect the bond angle N−Fe−N′ being 78.48(11)° in compound **1**, 78.61(14) and 78.04(14)° in [(dpp-BIAN)Fe^II^Cl_2_] [31], and 79.6(2) and 78.7(2)° in [(dpp-BIAN)Fe^II^Br_2_] [32]. The Fe−N bonds in complex **1** (2.128(3) and 2.139(3) Å are comparable to those in [(dpp-BIAN)Fe^II^Cl_2_] (range from 2.122(4) to 2.140(4) Å) [31] and slightly shorter than those in [(dpp-BIAN)Fe^II^Br_2_] (range: 2.131(6)–2.153(6) Å)) [32]. As a redox-active ligand, the dpp-BIAN can accept electrons, which should lead to a change in the α-diimine bond lengths [42,44]. In turn, an analysis of these bond lengths may suggest the ligand oxidation state. Inspection of the C−C and C−N bond lengths in the α-diimine fragment of compound **1** (C(1)−C(5): 1.506(5) Å; C(1)−N(1): 1.288(4) Å; C(5)−N(2): 1.284(4) Å), in free dpp-BIAN (C−C: 1.526(3) Å; C−N: 1.250(6) and 1.295(6) Å) [45], confirms a double bond character for C-N bonds in the dpp-BIAN ligand. On the other hand, bond length distribution in non-innocent complexes is considered as a necessary but not sufficient criterion for determining the electronic state of a ligand [46,47]. The use of a set of complementary methods is a common procedure for investigating the electronic structure of such systems.

The crystal structures of **1** at 100 and 280 K are isostructural (Table 1), changes in bond lengths and angles are insignificant (Table 2).

### 2.3. UV–VIS Spectra and Calculations

**Figure 2:**
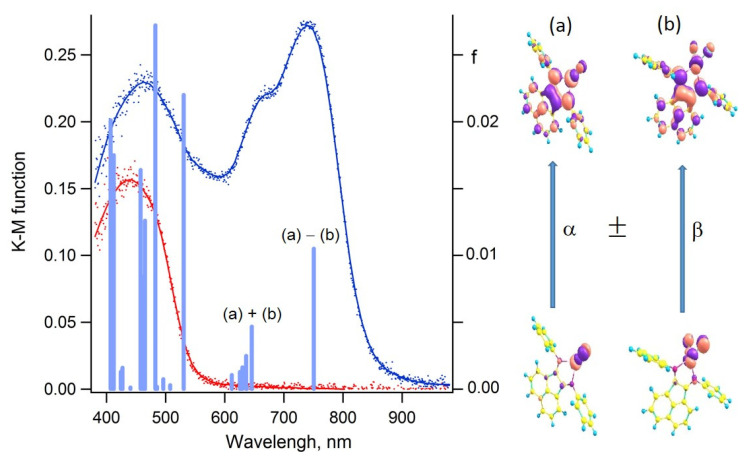
(**Left**) Solid-state electronic absorption spectra in the form of Kubelka–Munk function of dpp-BIAN (red spectrum) and its complex with FeI_2_ (blue spectrum); vertical blue bars indicate the positions and oscillator strengths (f, right axis) of the electronic transitions calculated at the B2PLYP/def2-TZVP level for complex [(dpp-BIAN)FeI_2_]. (**Right**) The electron promotions ((**a**,**b**)), which contribute mainly to the intense transitions at 751 ((a) − (b)) and 646 ((a) + (b)) nm.

The diffuse reflectance spectra of free dpp-BIAN and complex **1** were recorded in the solid state (polycrystalline sample dispersed in the BaSO_4_ matrix under argon atmosphere) and converted to the UV–VIS spectra (Figure 2). Figure 2 shows that the UV–VIS spectrum of **1** has an intense absorption band in the range of 450–550 nm, the same as in dpp-BIAN, and a broad intense band (λ_max_ = 740 nm) with a shoulder (at 660 nm) in the range of 600–850 nm. Two electron transitions contribute mainly to the latter band, and both of them are combinations (sum and difference) of promoting α- and β-electrons from MOs localized on FeI_2_ to π*-MOs of the dpp-BIAN ligand. Thus, this band is of the (M + X)LCT (metal-to-ligand and halide-to-ligand) type.

### 2.4. Solid State Magnetic Properties and Calculations

Before investigating the magnetic properties, it is very important to establish the oxidation state of Fe ion and the stability of complex **1** under ambient conditions. The purity of complex **1** has been determined by powder X-ray diffraction (Figure S2, SI), its chemical stability, and the Fe(II) oxidation state have been additionally confirmed by recording only Fe^2+^ lines in the Mössbauer spectrum (Figure S3, SI).

To understand magnetic properties of polycrystalline samples of **1** on a molecular level, we performed also high-level ab initio calculations (at both the relativistic CASSCF/NEVPT2 and non-relativistic CASCI levels) of the electronic structure and properties of complex **1** (for details, see SI). It was established that complex **1** has a quintet ground state (S = 2) with negligible contribution (<1%) of the configurations with occupation of the dpp-BIAN virtual MOs. The next four excited states are also quintet states, which have substantially higher energy (Figure S4a, SI) and are not occupied in the investigated temperature range. The zero-field splitting (ZFS) of the ground state was also predicted with a negative D value (from −7.8 to −16.7 cm^−1^ at different levels) and a moderate E/D ratio. At different levels of theory, the g_iso_ value is predicted to be in the range of 2.13–2.20 (Table S2, SI).

Thus, the results of Mössbauer and diffuse reflectance spectroscopies, as well as quantum chemical calculations, unambiguously prove the Fe(II) oxidation state and the absence of electron transfer between Fe(II) and dpp-BIAN during complexation, leading to the formation of [(dpp-BIAN)Fe^II^I_2_] complex. Since the value of D for **1** is negative, single-ion magnetic (SIM) behavior is possible for this complex. Therefore, the magnetic behavior of this complex on direct and alternating current was studied.

**Figure 3:**
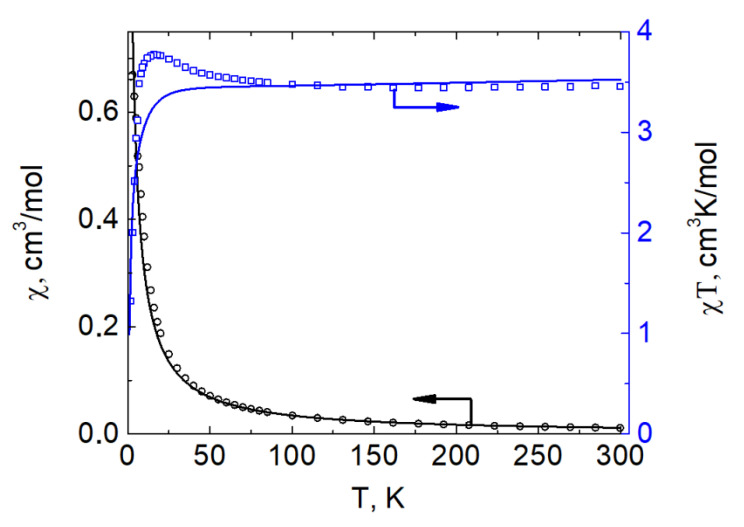
Experimental χ_m_ vs. T (black circles) and χ_m_T vs. T (blue squares) dependences for the complex **1** under 5000 Oe dc-field and corresponding theoretical curves calculated using the results of the SA-CASSCF(6,10)/NEVPT/SOC-QDPT calculations for 5 quintet and 45 triplet states.

The dc magnetic behavior of **1** was investigated under 5000 Oe dc magnetic field in the temperature range of 2–300 K. The experimental temperature dependences of the molar magnetic susceptibility (χ_m_) in the form of χ_m_ vs. T and χ_m_T vs. T are shown in Figure 3. As can be seen, the experimental χ_m_T values in the range of 200–300 K are close to the theoretical ones for the corresponding non-interacting complexes with S = 2 and g_iso_ = 2.15. This is due to the fact that the magnetic behavior of complex **1** is provided only by the contribution of Fe^2+^, since all ligands are diamagnetic. Figure 3 also displays the theoretical temperature dependences for non-interacting complexes **1**, calculated using the results of high-level quantum chemical calculations. For both the experimental and theoretical dependences, the χ_m_T values remain practically constant upon cooling from 300 to 100 K and close to each other. However, with a subsequent temperature lowering, the growth of the experimental χ_m_T values was observed with a maximum of 3.77 cm^3^ mol^−1^ K at 16 K followed by a sharp decrease to χ_m_T = 1.32 cm^3^ mol^−1^ K at 2 K. On the contrary, the theoretical χ_m_T value decreases monotonically with decreasing temperature from 100 to 2 K, reaching a value 1.83 cm^3^ mol^−1^ K at 2 K. The monotonic decrease in the theoretical dependence of χ_m_T vs. T is associated with the depopulation of the magnetic sublevels of the ground quintet state of Fe(II) (Figure S4b, SI). The presence of the maximum on the experimental curve can be explained by the reorientation of crystals of **1** under the 5000 Oe dc-field. Comparison with the theoretical curve suggests that the sharp decrease in the experimental χ_m_T at the low temperatures is mainly due to the depopulation of the magnetic sublevels of Fe^II^ ions.

In order to investigate single ion molecule magnet behavior, we performed magnetic measurements of [(dpp-BIAN)Fe^II^I_2_] in alternating current (ac) magnetic field, but there was no evidence of slow relaxation of the magnetization.

### 2.5. Cyclic Voltammetry and EPR Spectroscopy

It is well-known that dpp-BIAN can be reversibly reduced by alkali metals; it can accept stepwise from one to four electrons [42]; dpp-BIAN can exist in radical-anion or dianion form conjugated with a metal center (Ln or d-elements) [39,44,48,49,50,51]. However, electrochemical properties of the BIAN-complexes are not sufficiently investigated. Redox properties of several BIAN derivatives [52,53], as well as those of various α-diimine complexes of iron [30,54] have been reported. In order to enrich the level of discussion, we have studied the electrochemical behavior of the new complex **1** both in the solid state and in solution.

**Figure 4:**
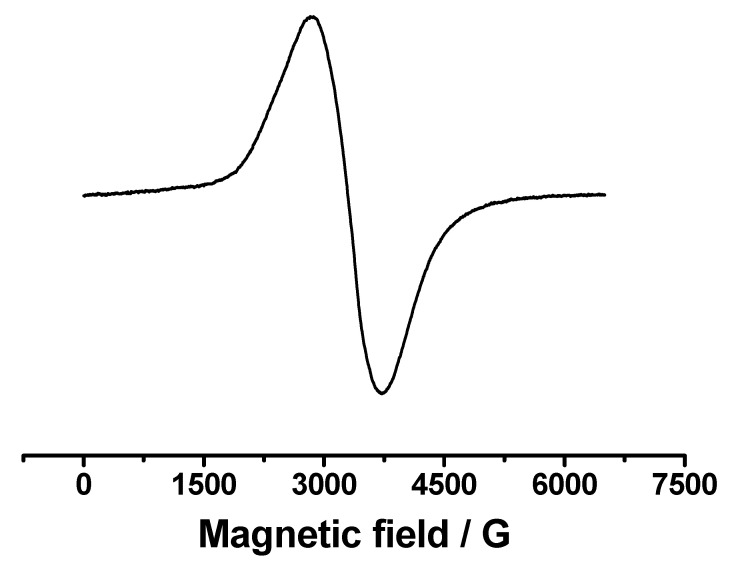
ESR spectrum of **1** powder, g = 2.10 and ∆H = 900 G.

The initial powder of **1** has an EPR signal, which is a broad line with parameters: g = 2.10 and ∆H = 900 G (Figure 4). Unfortunately, this information itself is not enough to unequivocally assign this spectrum to definite paramagnetic center. However, we can definitely say that the spectrum is associated with paramagnetic iron ions, and not with organic radicals in the composition of the sample. In contrast to solid state (Figure 4), solutions of complex **1** in acetonitrile and CH_2_Cl_2_ are EPR silent. This is not just a decrease in the signal intensity due to a multiple decrease in the concentration of the complex in solution as compared to its content in the powder, but it is indeed the disappearance of the EPR signal. The EPR silence of **1** in solution is associated with a rather large ZFS in the paramagnetic ground state of this complex. Thus, we can propose that the EPR spectrum in Figure 4 belongs not to the paramagnetic Fe ions of individual complexes **1**, but to the system of interacting paramagnetic Fe ions in the solid state. On the whole, this assumption is consistent with the unusual magnetic properties of **1**.

**Figure 5:**
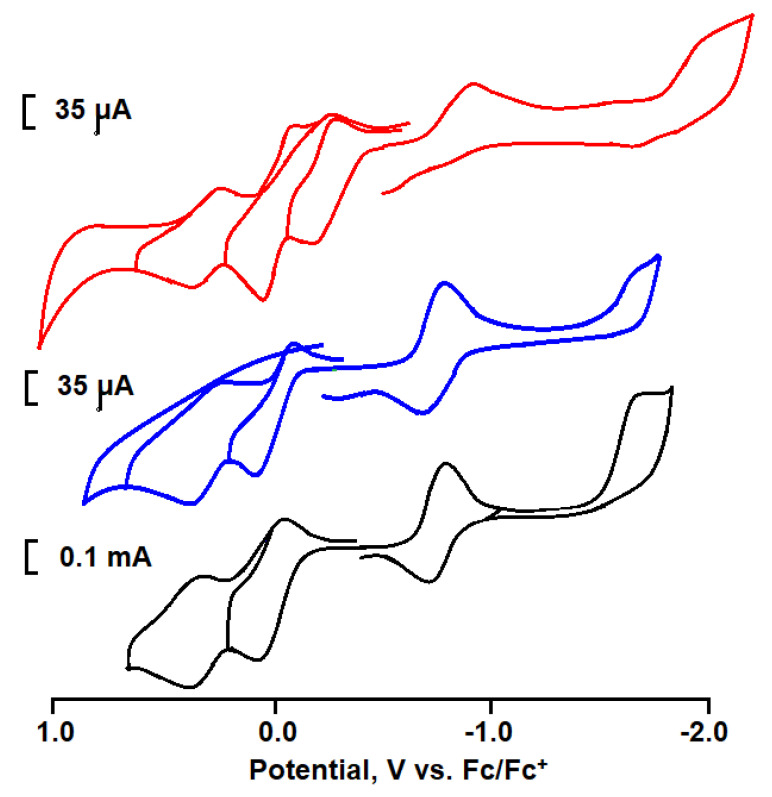
Cyclic voltammograms (CVs) of [(dpp-BIAN)Fe^II^I_2_]. **Black lines:** WE: CPE (graphite + ionic liquid + [(dpp-BIAN)Fe^II^I_2_]); **blue lines:** WE: GC; CH_2_Cl_2_; and **red lines:** WE: GC; CH_3_CN, 10^-1^ M Bu_4_NBF_4_ potentials vs. Ag/AgCl recalculated to Fc/Fc^+^, 100 mV/s.

**Scheme 2:**
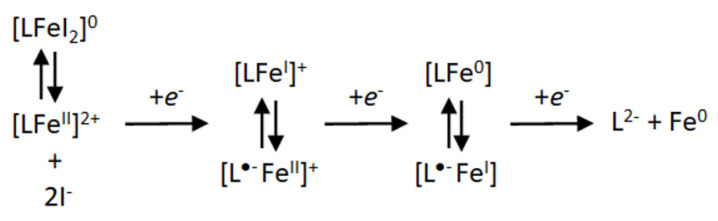
Cathodic transformation of complex **1**.

The voltammetric study of [(dpp-BIAN)Fe^II^I_2_] was carried out in two solvents (acetonitrile and CH_2_Cl_2_) as well as in the solid state [55] (Figure 5 and Table 3). The electrochemical behavior of **1** does not differ from that for the previously described chloride analogue [(dpp-BIAN)Fe^II^Cl_2_] [54,56] and, thus, can be explained by Scheme 2. It should be noted that the electrochemical behavior of **1** in the solid state and in the methylene chloride solution is the same, which indicates the sufficient stability of the complex in this solvent. The electrochemical behavior in the acetonitrile solution is different when the complex is dissolved in an inert gas atmosphere, a new characteristic reoxidation peak appears on the voltammogram (^1^E_p_a = −0.21 V).

**Figure 6:**
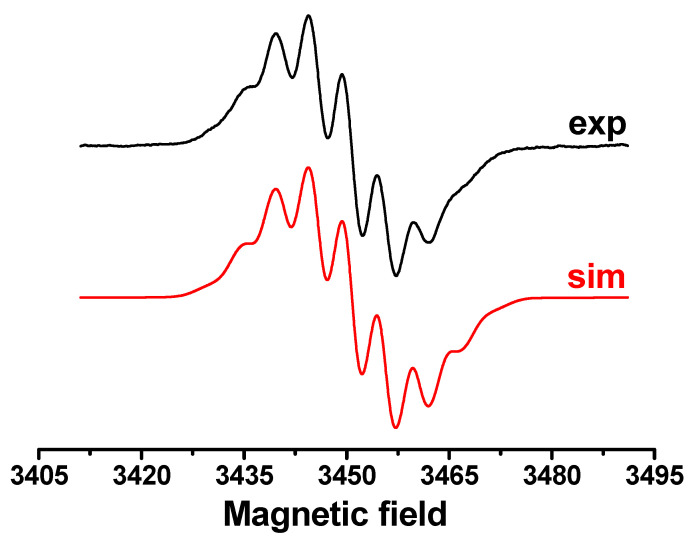
The EPR spectrum (black) of complex generated by cathodic reduction of [(dpp-BIAN)Fe^II^I_2_] in CH_2_Cl_2_ at −1.65 V vs. Fc/Fc+ and its simulation (red) with g = 2.0027, 2:a_N_ = 4.8 G, and 4:a_H_ = 4.7 G.

Using the EPR spectroelectrochemistry for the CH_2_Cl_2_ solution of **1**, the EPR signal was recorded at a potential of −1.65 V (Figure 6). The value of the g-factor (g = 2.0027) and splitting on two nitrogen nuclei (2:a_N_ = 4.8 G) indicates that we are dealing with the dpp-BIAN radical. Noteworthy is the presence of additional splitting at 4 protons (4:a_H_ = 4.7 G), which is not observed for the radical anion and radical trianion of the free dpp-BIAN. Thus, it is most likely that the reduction of the product of **1** in solution leads to the paramagnetic hetero-spin complex [(dpp-BIAN)^•−^Fe^I^].

## 3. Materials and Methods

### 3.1. General Remarks

All the synthetic procedures were carried out under a vacuum using glass ampules or under an argon atmosphere with standard Schlenk techniques. Acetonitrile was dried with P_2_O_5_, kept on the activated molecular sieves (3 Å), toluene was refluxed over sodium with benzophenone—both solvents were distilled under vacuum prior to use. The dpp-BIAN was synthesized by the condensation of acenaphthenequinone with 2,6-diisopropylaniline (both from Aldrich) under reflux in acetonitrile with a catalytic amount of acetic acid. Iron (II) iodide was synthesized every time in situ from carbonyl iron and iodine (both from Aldrich) in acetonitrile under heating in a Schlenk ampule with a Teflon stopcock. The yield of the product was calculated from the amount of the dpp-BIAN (0.50 g, 1.0 mmol) used in the syntheses. IR spectra were recorded on a Perkin Elmer Spectrum 65 spectrophotometer equipped with a Quest ATR Accessory (Specac) by the attenuated total reflectance (ATR) in the range of 400–4000 cm^−1^. Elemental analysis was performed on a Euro EA-3000 (Euro Vektor) automated C,H,N,S-analyzer. The powder patterns were measured using Bruker D8 Advance (λ(CuKα) = 1.5418 Å, Ni filter, Bragg-Brentano geometry) diffractometer. The obtained patterns were Rietveld refined using TOPAS 5 software. The diffuse reflectance data for a mixture of powder of **1** (or free dpp-BIAN) with BaSO_4_ were converted into the electronic absorption spectra applying a Kubelka–Munk (K-M) function [57].

The X-ray diffraction data sets for crystal of **1**·CH_3_CN were collected using Bruker SMART APEX II diffractometer equipped with a CCD detector (Mo-K_α_, λ= 0.71073 Å, graphite monochromator) [58]. Semiempirical absorption correction for **2** was applied [59]. The structure was solved by direct methods and refined by the full-matrix least squares with anisotropic displacement parameters using the SHELX-2014 program package [60]. The hydrogen atoms of the ligands were positioned geometrically and refined using the riding model. The structure parameters were deposited with the Cambridge Structural Database (CCDC Nos. 2077891 (**1**, 100 K) and 2077892 (**1**, 280 K); deposit@ccdc.cam.ac.uk or http://www.ccdc.cam.ac.uk/data_request/cif (accessed on 16 April 2021)).

Voltammograms of the [(dpp-BIAN)Fe^II^I_2_] complex were recorded in dry acetonitrile and CH_2_Cl_2_. A recrystallized n-Bu_4_NBF_4_ sample was placed in a Schlenk ampoule, it was evacuated and filled with argon. For this, 15 mL of acetonitrile was added through the septum. Then, 5 mL of the resulting solution was taken with a syringe to fill the electrochemical bridge. A portion of the complex was transferred under argon flow into a Schlenk vessel with n-Bu_4_NBF_4_ solution and dissolved under the influence of ultrasound. The resulting solution in argon flow was transferred by syringe into an electrochemical cell filled with argon.

DC-Magnetic susceptibility measurements were performed using a Quantum Design PPMS-9 susceptometer, under 5000 Oe magnetic field in the 2–300 K temperature range on polycrystalline sample sealed in polyethylene bag. The paramagnetic components of the magnetic susceptibility χ were determined taking into account both the diamagnetic contribution evaluated from Pascal’s constants and the contributions of the sample holder.

### 3.2. Synthesis of [(dpp-BIAN)Fe^II^I_2_]·CH_3_CN (1·CH_3_CN)

To a colorless solution of iron(II) iodide which was prepared in situ from iodine (0.254 g, 1.0 mmol) and an excess of iron powder in acetonitrile (25 mL) was added dpp-BIAN (0.500 g, 1.0 mmol). The reaction mixture was heated at 110 °C for 1 h in a sealed ampule. Colorless solution became dark-brown. Then, the reaction mixture slowly cooled (by 10 °C in an hour) to RT. A product started to crystallize at 50 °C. Big brown cubic crystals were isolated (0.82 g, 96%) after 24 h. Anal. Calcd for C_38_H_43_FeI_2_N_3_ (851.40) C, 53.61; H, 5.09; N, 4.94. Found: C, 53.28; H, 4.96; N, 4.78. IR, ν/cm^–1^: 2962 s, 2925 m, 2865 w, 2249 w, 1645 w, 1627 w, 1597 m, 1585 m, 1489 w, 1462 s, 1435 s, 1420 m, 1383 m, 1323 m, 1289 vs, 1252 m, 1222 w, 1203 w, 1187 m, 1129 m, 1088 w, 1052 m, 1041 m, 951 w, 936 w, 832 vs, 800 vs, 778 vs, 757 vs, 737 w, 624 w, 611 w, 587 w, 540 s, 512 w, 467 m, 441 w, 429 w, 420 w.

### 3.3. Details of Quantum Chemical Calculations

The positions and oscillator strengths of electronic transitions in the UV–VIS spectrum of **1** were calculated using time-dependent (TD) DFT [61] at the TD-UB2PLYP/def2-TZVP level [62].

The state-averaged (SA) CASSCF/NEVPT2 procedure [63,64] was used for the calculation of the wave functions of spin-free states. The scalar relativistic effects were taken into account using standard second-order Douglas−Kroll−Hess (DKH2) procedure [65,66]. The segmented all-electron relativistically contracted version of Ahlrichs polarized basis set def2-TZVP was used [67]. The active space for most of calculations (6, 10) consisted of six electrons distributed on five 3d-orbitals and five double-shell d-orbitals [68]. The fine structure of ground and excited states was computed with the Breit-Pauli approximation and the mean field approximation (SOMF) [69]. Five quintet and 45 triplet states of Fe^II^ were included. Spin Hamiltonian (SH) parameters and g tensors have been computed with an effective Hamiltonian (QDPT) [70]. All calculations were performed with the ORCA 4.1.1 computational package [71].

The CASSCF/NEVPT2 wavefunctions of complex **1** were also used to calculate temperature dependence of its molar magnetic susceptibility. In these calculations, both the SOC and Zeeman effects were taken into account. Magnetic susceptibility of each magnetic sublevel was computed numerically as the second derivative of the energy with respect to the magnetic field. The molar magnetic susceptibility is computed as averaged according to Boltzmann statistics. In order to reproduce powder measurement, the averaging over sphere for directions of magnetic field has been performed. Described treatment, realized in the ORCA package [71], takes into account mixing of states due to both the spin−orbit coupling and magnetic field up to infinite order of perturbation theory.

## 4. Conclusions

Here, we reported on a novel molecular iodide iron(II) complex with neutral 1,2-bis[(2,6-diisopropylphenyl)imino]acenaphthene, [(dpp-BIAN)Fe^II^I_2_]. It is known that dpp-BIAN can exist in a radical-anion or dianionic form conjugated with a metal center, which were previously isolated [50]. In this paper, based on the data of single crystal X-ray diffraction, we proved the absence of redox isomerism in the solid state and the preservation of an almost identical crystal structure of **1** in the temperature range of 100–300 K. The EPR spectrum of solid sample of **1** is associated with paramagnetic Fe ions, and not with organic radicals in the composition of the sample. The oxidation state of the Fe(II) ion in **1** was confirmed by Mossbauer spectroscopy, magnetic susceptibility measurements, and high-level quantum chemical calculations. The neutral form of dpp-BIAN is confirmed by the features of the IR and UV–VIS spectra. Thus, according to comprehensive experimental studies and ab initio calculations, solid sample of **1** consists of an iron(II) ion and a neutral form of a non-innocent ligand.

Cyclic voltammetry of **1** demonstrated that the transition of the dpp-BIAN ligand to the anion-radical form occurs in two steps: (1) reduction of the iron(II) ion into iron(I) ion and (2) reduction of the ligand. The electrochemical behavior of complex **1** in the solid state and in methylene chloride is the same. The product of two-electron reduction in a CH_2_Cl_2_ solution (E_p_ = −1.65 V vs. Fc/Fc+) was detected by EPR method and assigned to the paramagnetic hetero-spin complex [(dpp-BIAN)^•−^Fe^I^].

## Figures and Tables

**Table 1:** Crystal data and structure refinement details for compound **1**·CH_3_CN.

	**1**·CH_3_CN	
Formula	C_38_H_43_FeI_2_N_3_	
Mr (g mol^−1^)	851.40	
T (K)	100	280
Crystal system	Orthorhombic	
Space group	*Pbca*	
a (Å)	19.400(2)	19.568(4)
b (Å)	19.303(3)	19.309(5)
c (Å)	19.581(2)	19.824(3)
V (Å^3^)	7333.0(18)	7490(3)
Z	8	
ρ_calc_,(g cm^−3^)	1.542	1.510
μ (mm^−1^)	2.124	2.080
F(000)	3392	3392
Crystal size, (mm)	0.12 × 0.12 × 0.04	
θ_min_/θ_max_ (°)	2.08/28.28	2.06/26.37
Index ranges	−25 ≤ h ≤ 25	−24 ≤ h ≤ 24
	−25 ≤ k ≤ 25	−24 ≤ k ≤ 24
	−26 ≤ l ≤ 26	−24 ≤ l ≤ 24
Reflections collected	75400	66864
Independent reflections	7386	4922
R_int_	0.0847	0.1118
T_min_/T_max_	0.6758/0.7461	0.6599/0.7461
GOF on F^2^	1.100	1.047
Final R indices (I > 2σ(I))	R_1_ = 0.0475, wR_2_ = 0.0792	R_1_ = 0.0690, wR_2_ = 0.1363
R indices (all data)	R_1_ = 0.0654, wR_2_ = 0.0847	R_1_ = 0.1167, wR_2_ = 0.1568
Largest diffraction peak/hole (e Å^−3^) (*d*_*min*_/*d*_*max*_)	−0.863/0.732	−0.885/0.685

**Table 2:** Selected bond lengths (Å) and angles (°) in **1**.

Temperature/Parameter	100 K	280 K
C (1)−C (5)	1.506 (5)	1.504 (8)
N (1)−C (1)	1.288 (4)	1.294 (7)
N (2)−C (5)	1.284 (4)	1.285 (7)
Fe (1)−N (1)	2.128 (3)	2.124 (5)
Fe (1)−N (2)	2.139 (3)	2.139 (5)
Fe (1)−I (1)	2.5654 (6)	2.5542 (10)
Fe (1)−I (2)	2.5683 (6)	2.5556 (11)
I(1) − Fe(1) − I(2)	113.44 (2)	112.79 (4)
N(1) − Fe(1) − N(2)	78.48 (11)	78.27 (17)
Fe…Fe_min_	9.747 (2)	9.755 (4)

**Table 3:** Peak potentials (V) for **1** (potentials vs. Fc/Fc^+^).

Compound	Solvent	Reduction	Oxidation
[(dpp-BIAN)Fe^II^I_2_]	CH_3_CN	−0.95/- −2.00/-	−0.21/−0.31 0.02/−0.13 0.33/0.21
	CH_2_Cl_2_	−0.82/−0.71 −1.67/-	0.05/−0.10 0.33/0.21
	Solid	−0.82/−0.76 −1.69/-	0.05/−0.03 0.35/0.27

## Data Availability

Not avalible.

## Supplementary Materials

The following are available online. Figure S1: IR spectrum of compound; Figure S2: Theoretical and experimental powder patterns of **1**, Figure S3: Mossbauer spectrum of compound **1**, Figure S4: the energies of the lowest spin-multiplets (S = 2) of complex **1** (a) and energies of magnetic sublevels of the ground quintet state, Figure S5: d-orbitals of Fe(II) in upper row and LUMO and LUMO+1 of dpp-BIAN ligand in **1**, Table S1: Mossbauer parameters of iron in compound **1**, Table S2: The fine structure and SH-parameters for the quintet ground states of complexes **1**.
