# Supplementary material for: Generation of a Hetero Spin Complex from Iron(II) Iodide with Redox Active Acenaphthene-1,2-Diimine
Source: Molecules. 2021 May 18;26(10):2998. doi: 10.3390/molecules26102998 (PMC8158106; doi:10.3390/molecules26102998)
Supplement: Supplementary file 1 [file molecules-26-02998-s001.zip › molecules-1205735-supplementary.pdf]

molecules

SUPPLEMENTARY INFORMATION

for the article

Generation of an hetero spin complex from iron(II) iodide with redox active acenaphtene-1,2-diimine

Dmitriy S. Yambulatov[1,*], Stanislav A. Nikolaevskii[1,*], Mikhail A. Kiskin[1], Kirill V. Kholin[2], Mikhail N. Khrizanforov[2], Yulia G. Budnikova[2], Konstantin A. Babeshkin[1], Nikolay N. Efimov[1], Alexander S. Goloveshkin[3], Vladimir K. Imshennik[4], Yurii V. Maksimov[4], Evgeny M. Kadilenko[5], Nina P. Gritsan[5] and Igor L. Eremenko[1]

1 N. S. Kurnakov Institute of General and Inorganic Chemistry, Russian Academy of Sciences, 31 Leninsky prosp., 119991 Moscow, Russian Federation. Fax: +7 (495) 952 1279. E-mail: yambulatov@yandex.ru (D.S. Y.); sanikol@igic.ras.ru (S.A.N.)
2 Arbuzov Institute of Organic and Physical Chemistry, FRC Kazan Scientific Center of RAS, Arbuzov Str. 8, 420088 Kazan, Russian Federation yulia@iopc.ru
3 Nesmeyanov Institute of Organoelement Compounds, Moscow, 119991, Russia golov-1@mail.ru (A.S.G.)
4 N. N. Semenov Institute of Chemical Physics, Russian Academy of Sciences, Kosygina Str. 4, 119991 Moscow, Russian Federation vladim_imshennik@mail.ru
5 V.V. Voevodsky Institut of Chemical Kinetics and Combustion, SB RAS, 3 Institutskaya str., 630090 Novosibirsk, Russian Federation gritsan@kinetics.nsc.ru
* Correspondence: yambulatov@yandex.ru (D.S.Y.); sanikol@igic.ras.ru (S.A.N.); Tel.: +7-915-955-2442 (D. Y.); +7-495-955-4817 (SN)

Supporting information

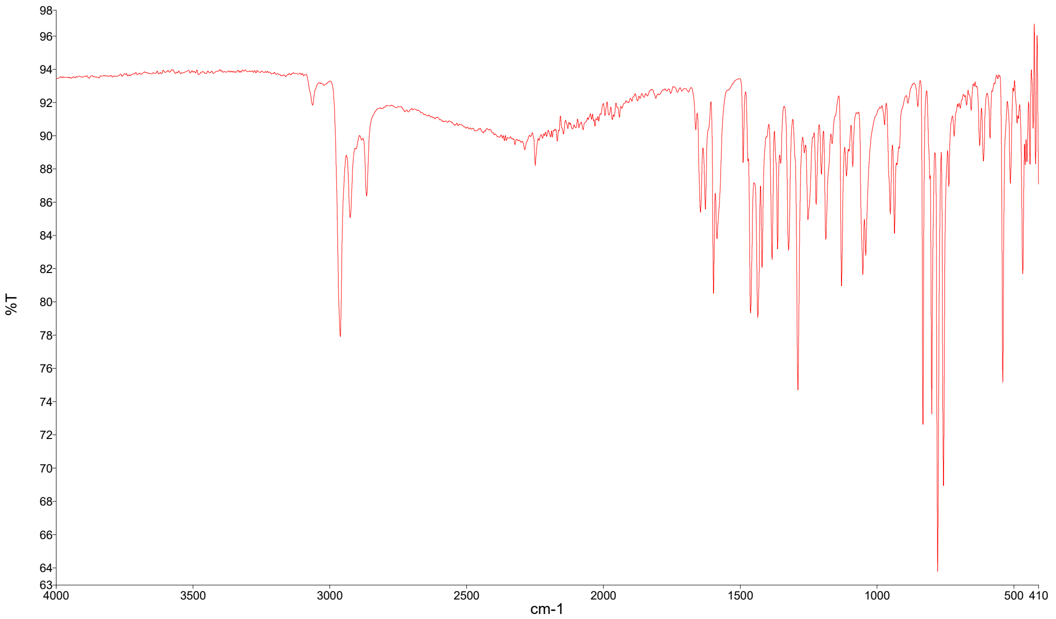

Figure S1. IR-spectrum of [(dpp-BIAN)$Fe^{II}I_2$].

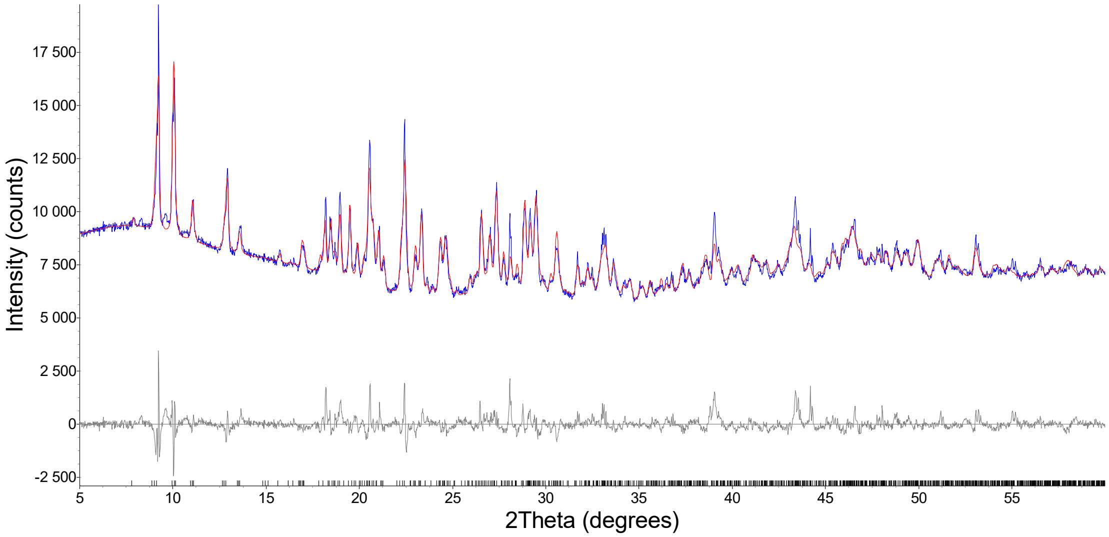

Figure S2. Theoretical (red line) and experimental (blue line) powder patterns of the [(dpp-BIAN)$Fe^{II}I_2$] and their difference (grey line).

*Mössbauer spectroscopy for [(dpp-BIAN)$Fe^{II}I_2$] (**1**)*

The Mossbauer measurements with ^{57}Fe were performed at 300 K (±0.1 K) using an electrodynamic-type spectrometer (Wissel, Germany) and a CCS-850 helium cryostat (Janis, USA) with a 332 temperature controller (Lake Shore Cryotronics, USA). The Mössbauer radiation source was ^{57}Co(Rh) with an activity of 1.1 GBq. The Mossbauer spectra were processed by standard least-squares programs (LOREN, IHF RAS, and NORMOS, Germany) under the assumption of the Lorentz line shape. Isomeric shifts were measured from the center of the magnetic hyperfine structure (HFS) of metallic iron.

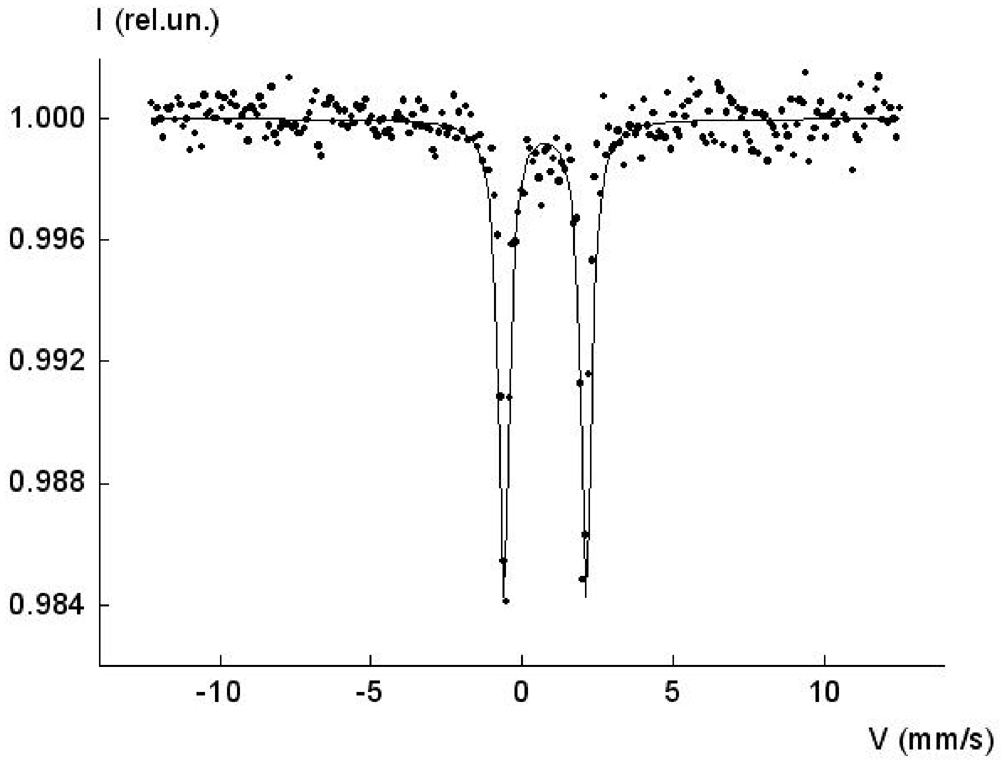

Figure S3. Mossbauer spectrum at T=300K of the sample [(dpp-BIAN)$Fe^{II}I_2$] (**1**).

Table S1. Mossbauer parameters of iron at T=300K of the sample [(dpp-BIAN)$Fe^{II}I_2$] (**1**).

Sample	Fe site	δ	Δ	H_{in},	Γ	A,
		±0.03 mm/s		±0.5T	±0.03 mm/s	±0.05
**1**	Fe^{2+} - paramagnetic	0.74	2.71	-	0.41	1.00

δ is the isomeric shift with respect to α-Fe; Δ is the quadrupole splitting or the quadrupole shift; H_{in} is the internal magnetic field on the nucleus ^{57}Fe, Γ is the line width, A is the relative content.

Quantum chemical calculation of spectroscopy and magnetic properties of complex **1***.*

To support experimental findings and to understand magnetic properties of complex **1** on a molecular level, we calculated the fine structure of its ground and excited states and parameters of the spin-Hamiltonian. Figure S4 shows the lowest-energy levels of **1**, all of them correspond to spin-multiplets with S = 2, which are split into magnetic sublevels, taking into account spin-orbit coupling (SOC) (Fig. S4b). Calculations of the spin multiplets were performed by the state-averaged (SA) CASSCF(6,10) level with 6 electrons on a double d-shell (10 MOs), dynamic electron correlations was accounted at the NEVPT2 level.

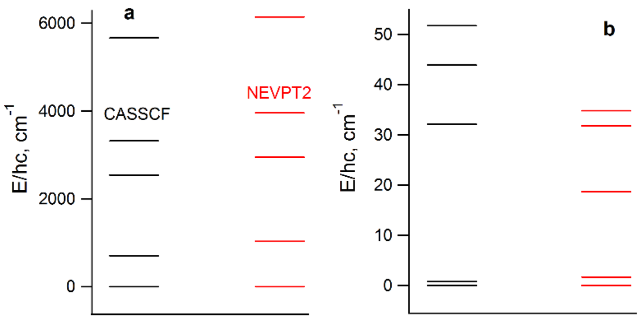

Figure S4. The energies of the lowest spin-multiplets (S = 2) of complex **1** (a) and energies of magnetic sublevels of the ground quintet state calculated at the SA-CASSCF/SOC-QDPT (black lines) and SA-CASSCF/NEVPT2/SOC-QDPT (red lines) levels with the scalar relativistic DKH2 Hamiltonian.

Table S2 represents the energy splitting for the ground quintet of **1** (Fig. S4b), as well as results of more simple calculations with non-relativistic Hamiltonian and account of SOC at CASCI level. Analysis of Table S2 demonstrates that both approaches predict similar fine structure and spin-Hamiltonian parameters for **1**. For both complexes, the quintet is a ground state with the first excited state lying much higher in energy: by 650 – 1040 cm^{-1} for **1** (depending of the level of theory). Thus, only sublevels of the ground quintet state are occupied in the experimental temperature range. The negative D value was predicted for **1** (Table S2) and the SIM properties are possible for this complex.

Table S2. The fine structure and SH-parameters for the quintet ground states of complexes **1** predicted at different levels of theory and presented in cm^{-1} and in K (in parenthesis).

Fine str., SH-par.	Relativistic calculations		CASCI/def2-TZVP (ECP for I)	
	CASSCF(6,10)	NEVPT2	CAS(6,10)	CAS(6,12)
E_0	0	0	0	0
E_1	0.8 (1.2)	1.7 (2.4)	1.2	0.1
E_2	32.1 (46.2)	18.7 (26.9)	40.6	45.6
E_3	43.9 (63.2)	31.8 (45.8)	56.4	52.1
E_4	51.7 (74.4)	34.8 (50.1)	66.4	66.6
D, E/D	-12.4, 0.16	-7.8, 0.28	-15.8; 0.17	-16.7; 0.06
g_x, g_y, g_z	2.01, 2.10, 2.36	2.02, 2.10, 2.26	2.00, 2.10, 2.39	2.06, 2.10, 2.45
g_{iso}	2.16	2.13	2.16	2.20

To check the innocence of dpp-BIAN ligand in **1**, the CASSCF calculations with a larger active space (6,12) with two additional orbitals (LUMO and LUMO+1 of the dpp-BIAN, Fig. S5) were also performed (Table S2). The contribution of configurations with occupation of the LUMO or LUMO+1 is minor (< 2%) in all states with the energy up to 21000 cm^{-1}. The excited states corresponding to the electron transfer from Fe(II) to the LUMO of dpp-BIAN lye at 21580 cm^{-1} (S = 3) and 24570 cm^{-1} (S = 2)

indicating the ferromagnetic interaction between Fe^{3+} and radical anion of the dpp-BIAN with J = 1500 cm^{-1}.

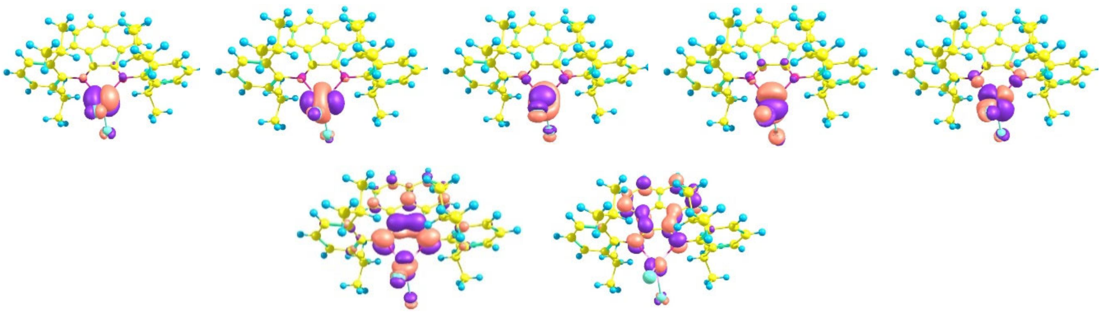

Figure S5. Seven out of twelve active orbitals (d-orbitals of Fe(II) in upper row and LUMO and LUMO+1 of dpp-BIAN ligand) involved in the CASSCF and CASCI calculations for complex **1** are presented (double-shell d-orbitals are not presented as their occupation is minor).
